# Strategic Use of Affiliative Vocalizations by Wild Female Baboons

**DOI:** 10.1371/journal.pone.0163978

**Published:** 2016-10-26

**Authors:** Joan B. Silk, Robert M. Seyfarth, Dorothy L. Cheney

**Affiliations:** 1 School of Human Evolution and Social Change, Arizona State University, Tempe, AZ 85287, United States of America; 2 Department of Psychology, University of Pennsylvania, Philadelphia, PA 19104, United States of America; 3 Department of Biology, University of Pennsylvania, Philadelphia, PA 19104, United States of America; University of Sussex, UNITED KINGDOM

## Abstract

Although vocal production in non-human primates is highly constrained, individuals appear to have some control over whether to call or remain silent. We investigated how contextual factors affect the production of grunts given by wild female chacma baboons, *Papio ursinus*, during social interactions. Females grunted as they approached other adult females 28% of the time. Supporting previous research, females were much more likely to grunt to mothers with young infants than to females without infants. Grunts also significantly increased the likelihood of affiliative interactions among all partners. Notably, however, grunts did not simply mirror existing social bonds. Instead, they appeared to perform a very different function: namely, to serve as signals of benign intent between partners whose relationship is not necessarily close or predictable. Females were less likely to grunt to their mothers or adult daughters—the individuals with whom they shared the closest and least aggressive bonds—than to other females. In contrast, patterns of grunting between sisters were similar to those between nonkin, perhaps reflecting sisters’ more ambivalent relationships. Females grunted at higher rates to lower-ranking, than to higher-ranking, females, supporting the hypothesis that grunts do not simply signal the signaler’s level of arousal or anxiety about receiving aggression, but instead function as signals of benign intent. Taken together, results suggest that the grunts given by female baboons serve to reduce uncertainty about the likely outcome of an interaction between partners whose relationship is not predictably affiliative. Despite their limited vocal repertoire, baboons appear to be skilled at modifying call production in different social contexts and for different audiences.

## Introduction

Social groups of animals contain individuals with different energetic needs, reproductive strategies, genetic interests, and competitive abilities. Nonetheless, group members are able to synchronize their activities, coordinate travel, and maintain social relationships that include both cooperative and competitive elements. Vocal signals play an important role in this process. In addition to signaling the presence of predators and food, vocalizations indicate callers’ readiness to travel or the direction in which they intend to move (e.g. [1–3]). Such calls enhance group-level coordination, even when group members have competing preferences. Some of the calls produced by monkeys also predict their likelihood of behaving peacefully. These calls can facilitate friendly social interactions or act as reconciliatory signals following aggression [4–9]. In some cases, call exchanges appear to function as long-distance ‘social grooming’, and to reinforce existing social relationships [10–11].

During the past decades, much has been learned about the function of calls for listeners (for reviews see [12–14]). We still know little, however, about the factors that cause an individual to vocalize or remain silent. By some measures, call production in primates and many other mammals is highly constrained. Primates, for example, have relatively small vocal repertoires of calls that are predictably linked to specific social contexts and show limited acoustic modification during development [15]. Other features, however, suggest some flexibility in call production. In laboratory tests, the timing, duration, and rate of calling can be brought under operant control [16]. Furthermore, there is evidence from both field and laboratory studies that callers can modify whether to call or remain silent [17] and the type of call given [18–19] depending upon auditory input or the presence, identity, or behavior of an audience (reviewed in [20]).

Female chacma baboons (*Papio ursinus*) sometimes give low amplitude grunts as they approach other females [6]. Listeners can recognize the identity of callers based on acoustic cues [21], and they appear able to infer whether they are the target of another individual’s grunts through the use of multiple contextual cues, including the vocalizer’s direction of gaze and the nature of recent interactions with the vocalizer and her close kin [14, 20, 22]. Grunts directed to lower-ranking females are effective in facilitating peaceful interactions, including grooming and infant handling [6, 23]. Subordinate females are less likely to move away from an approaching female when she grunts than when she remains silent [6]. Grunts also serve a reconciliatory function. Grunts by the aggressor shortly after a conflict are associated with a lower likelihood that the conflict will continue [7]. Experiments using playbacks of vocalizations show that grunts lower victims’ concern about being the target of redirected aggression by their former opponents [6] and also make it more likely that females will approach former aggressors after conflicts [5].

Several previous studies have suggested that the exchange of contact calls may function to reinforce individuals’ social bonds, especially when they occur between widely separated partners [10–11]. However, it is also possible that these calls are used to monitor the location of important social partners, not to reinforce their bonds. In playback experiments, chacma baboons selectively respond to their close relatives’ loud ‘contact’ barks [24]. Although chacma baboons also exchange grunts in the context of group travel [17, 21], it is not yet known whether such calls are exchanged selectively between close partners.

Here, we build on previous work and investigate how contextual factors affect the production of grunts in the context of social interactions among female chacma baboons. We first investigate the relationship between females’ grunting and other patterns of affiliative behavior. Female baboons establish strong and enduring bonds with close maternal kin (mothers, daughters, and sisters)[25–29]. Relationships between mothers and daughters are characterized by particularly high rates of affiliation and low rates of conflict, while relationships between maternal sisters are simultaneously affiliative and competitive [28]. If grunting primarily serves to reinforce social bonds, we would expect females to grunt at high rates to close kin and at low rates to non-kin. In contrast, if grunts function to reduce uncertainty about the signaler’s likely behavior, we would expect females to grunt at comparatively lower rates to their mothers and daughters, with whom the outcome of any interaction is highly predictable, and at higher rates to their sisters and nonkin, with whom the outcome of any interaction is less certain.

We next consider females’ grunting patterns toward mothers with infants. Female baboons are strongly motivated to interact with infants [23, 30, 31], but such initiations can lead to different outcomes depending on the relative ranks of the females involved. Mothers will often threaten an approaching lower-ranking female, but avoid and move away from a higher-ranking one. Grunts appear to facilitate these interactions, and most approaches toward mothers are accompanied by high rates of grunting [9, 23]. Females often appear to be highly aroused when approaching mothers with infants, as suggested by changes in the temporal and spectral patterns of their grunts [32]. If grunting is motivated primarily by the signaler’s anxiety about a possible aggressive response from the mother, we would expect females to grunt at higher rates when approaching mothers that are higher-ranking than they are, and at lower rates when approaching mothers that are lower-ranking. Conversely, if approaching females are attempting to reassure potential partners about their benign intentions, they should grunt at higher rates to mothers that are lower-ranking, and at lower rates to mothers that are higher-ranking than they are.

## Methods

### Study Group

Data were derived from a long-term study of wild chacma baboons (*Papio ursinus*) in the Moremi Game Reserve, Botswana [33]. The study group was habituated in 1978 by W. J. Hamilton and colleagues. From June 1992 through December 2007, the study group was observed almost daily. As in other species of cercopithecine primates, female baboons are philopatric and assume dominance ranks similar to their mothers’ [25–27]. Previous analyses of the study group have shown that female baboons’ social relationships are highly differentiated and biased toward maternal kin. Females form the strongest and most enduring bonds with their mothers and daughters. Bonds between maternal sisters tend to be weaker than those between mothers and daughters, but significantly stronger than bonds between less closely related females [28].

The analyses presented below are based on focal animal samples collected during 1992–93 and 2001–2007. During this period, the group contained on average 77.6 individuals (range: 65–88), and included on average 26.4 adult females (range 23–31), defined as those >5 years of age, and 10.4 adult males (range 9–12), defined as natal males > 9 years of age and all immigrant males. Maternal kinship was known for all individuals. The primary causes of mortality were infanticide and predation.

### Behavioral data

Ten-minute focal animal observations [34] on all adult males and females were conducted almost daily using a common protocol. We recorded all approaches (to within 2 m), vocalizations, and friendly and aggressive interactions on a continuous basis. This sample included 12,263 approach sequences involving 64 adult females. We also noted all grooming interactions and their durations. Adult female dominance ranks were calculated monthly based on the direction of approach-retreat interactions [25]. For most of the study, the female dominance hierarchy remained stable.

We also used focal animal samples to calculate rates of social interactions. Rates of social interactions were computed by dividing the number of each type of event by the amount of time observed. The proportion of time spent grooming was computed by dividing the summed durations of grooming within each dyad by the total amount of time observed.

Following previous work [28, 35–37], we calculated composite dyadic sociality indices (DSI) for every female dyad. The DSI is based on the relative frequencies of positively correlated non-aggressive social interactions, and measures the extent to which each dyad deviates from other dyad. For this population, the DSI is based on the rate of approaches, groom presents, grooming initiations, and the duration of grooming within dyads (both given and received) [35].

### Analysis

We created a database that included each approach, the identity of the individual who initiated the approach (the actor) the identity of the individual who was the recipient of the approach (her partner), and subsequent vocalizations and social interactions involving the actor and her partner that occurred within the next minute (usually within several seconds). For each approach sequence, we identified the first two vocalizations or social behaviors that occurred involving the actor and the partner. For the purposes of these analyses, we categorized social behaviors into three categories: affiliation, aggression, and infant handling. Affiliative behaviors included groom, present, inspect, embrace, lip smack, and peaceful contact. Aggressive behaviors included threats and contact aggression. Infant handling included all behaviors directed toward the partner’s infant.

For analyses with binary outcome variables we used multi-level mixed effect logistic regression models and for analyses with continuous outcome variables we used multi-level mixed effect linear regression models. We treated kinship, relative rank, and the presence of infants as a categorical variables (nonkin = 0, cousins = 1, aunts and nieces = 2, sisters = 3, mothers and daughters = 4). Relative rank was scored as 1 if the approaching female was higher-ranking than the female she approached and 0 if the approaching female was lower-ranking than the female she approached. Infant presence was scored as 1 if the female who was being approached had an infant under the age of 6 months, and 0 if she did not. Pairs of females were involved in different numbers of approach sequences, so we treated dyad as a random effects variable in the analyses. All analyses were conducted with STATA 11 or R version 3.1.2.

## Results

### Frequency and consequences of grunting

Females grunted to their partner in 28% (n = 12263) of all approaches. The presence of young infants (< 6 months of age), relative rank, and kinship all influenced the likelihood that a female would grunt as she approached another female (Table 1). Controlling for other predictors (relative rank, maternal kinship), females were approximately 14 times more likely to grunt to a female with an infant than they were to grunt to a female without an infant. Females rarely exchanged grunts during social interactions, unless both partners had young infants.

**Table 1:** Factors that influenced whether females grunted after they approach another female.

	Odds Ratio	S.E.	Z	P
Infant presence	14.47	0.60	63.94	< 0.001
Relative rank	1.41	0.08	6.45	<0.001
Kinship				
Cousins	1.07	0.24	0.28	0.777
Aunts & nieces	0.94	0.12	-0.51	0.608
Sisters	0.96	0.14	-0.29	0.770
Mothers & daughters	0.48	0.07	-4.89	< 0.001

Results were obtained from a multi-level mixed-effects logistic regression model (n = 12,263 approaches involving 64 adult females).

**Fig 1:**
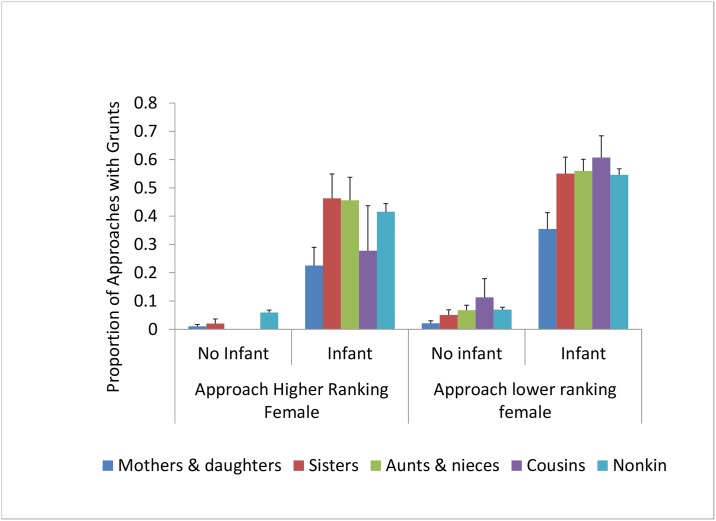
The proportion of approaches that were accompanied by grunts. Females grunted as they approached other females 28% of the time overall.

Overall, individuals were more likely to grunt when approaching lower-ranking females than when approaching higher-ranking females (Fig 1). This pattern was evident both when infants were present (Odds Ratio: 2.78, z = 32.27, p = 0.001) and when infants were absent (Odds Ratio: 1.05, z = 2.93, p = 0.003).

Maternal kinship also affected the likelihood that an approaching female would produce grunts (Table 1). Females were only half as likely to grunt when they approached their mothers or daughters as when they approached unrelated females (Fig 1). In contrast, females were as likely to grunt to their sisters as they were to unrelated females (Fig 1).

Sisters whose relationship was characterized by high rates of aggression were significantly more likely to grunt when approaching each other (Table 2). There was no relation, however, between sisters’ grunting frequencies and their DSI. Grunting frequencies between mothers and daughters was unrelated to either their rate of aggression or the strength of their relationship (Table 2).

**Table 2:** The relation between grunting frequencies and rates of aggression and DSI for (a) mothers and daughters and (b) maternal sisters.

	Estimate (β)	S.E.	z	P
**(a) Mothers/daughters**				
Aggression rate	0.193	0.306	0.633	0.527
DSI	-0.029	0.046	-0.632	0.527
Aggr. x DSI interaction	0.053	0.055	0.967	0.334
**(b) Sisters**				
Aggression rate	0.313	0.134	2.340	0.019
DSI	0.060	0.101	0.593	0.553
Aggr. x DSI interaction	-0.047	0.031	-1.515	0.130

Results were obtained from a multi-level mixed effect logistic regression model in which the rate of aggression and DSI served as predictor values and the likelihood of grunting (yes/no) during an approach was the dependent variable.

### Subsequent behavior

Grunts were effective in facilitating infant handling and affiliation. Females who grunted as they approached a mother with a young infant were 48 times more likely to handle their partners’ infants than females who remained silent (Table 3). Similarly, females were four times more likely to engage in affiliative interactions like grooming if they grunted as they approached than if they did not grunt. Finally, grunts were also associated with a lower likelihood that the caller would behave aggressively. Females who remained silent as they approached were eight times more likely to behave aggressively than females who grunted. This result was not altered when the analysis was limited to sequences in which females approached lower-ranking females.

**Table 3:** Effects of grunts on likelihood of subsequent interactions.

	Odds Ratio	S.E.	Z	P
Infant handling	47.66	3.89	47.60	< 0.001
Affiliation	4.08	0.23	24.50	< 0.001
Aggression	0.12	0.05	-4.70	< 0.001

Analysis controlled for kinship and relative rank.

**Fig 2:**
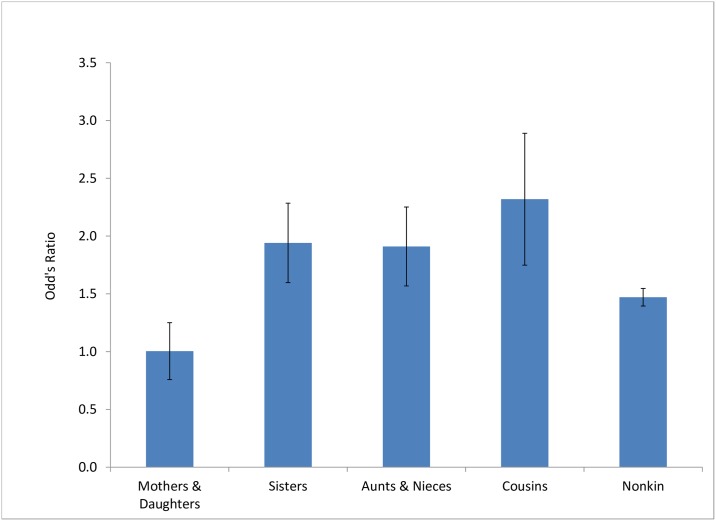
The effects of actors’ grunts on the likelihood of subsequent affiliation with kin and nonkin. Effects of the presence of infants and relative rank were controlled in each model. Bars show standard errors.

Grunting had no effect of on the likelihood of affiliation between mothers and daughters. For nonkin and all other categories of maternal kin, however, grunting substantially increased the likelihood of affiliation (Fig 2).

## Discussion

Females grunted as they approached other females in more than one quarter of their approaches. Females were more likely to grunt if their partner was lower-ranking than themselves or had a young infant. On the other hand, they were substantially less likely to grunt when they approached their mothers and daughters than when they approached others. Taken together, these results that female baboons use grunts strategically—grunting when calls play an important role in facilitating social interactions and remaining silent when such calls have little impact or when there is less uncertainty about the probable outcome of an interaction.

If grunts reinforce social bonds, then mothers and daughters would be expected to grunt more than other pairs of females, because they have the closest social bonds [25, 28]. Instead, the low likelihood of grunting among mothers and daughters may reflect the fact that grunts play a smaller role in facilitating their interactions than among other pairs of females. In the absence of grunts, mothers and daughters were more likely to behave affiliatively and less likely to behave aggressively than were other pairs of females, perhaps mitigating the need for signals of benign intent.

This pattern, however, held only for mothers and daughters. Notably, patterns of grunting between sisters were similar to those among nonkin. Although social bonds between sisters are significantly stronger than those between less closely related individuals, their rates of aggression are similar to those among nonkin [28]. Thus, when one sister approaches another there is some uncertainty about whether her subsequent behavior will be friendly or aggressive. Grunts to sisters, like grunts to unrelated females, may function to reduce uncertainty by signaling the approaching sister’s low likelihood of aggression. Supporting this hypothesis, sisters whose relationship was more aggressive were more likely to grunt to each other than sisters whose relationship was less aggressive.

Prior research has suggested that the acoustic features of baboon grunts are affected by the caller’s affect, or level of arousal [32]. Although human judgments about an animal’s arousal are inevitably subjective to some degree, we can speculate that a female is more excited when she is approaching a partner with an infant than without one and more anxious when approaching a higher-, rather than a lower-ranking, partner. Supporting one of these predictions, we found that grunting was indeed more likely when infants were present. However, we also found that females were more likely to grunt to lower-ranking partners than to higher-ranking partners. Thus, affect and level of arousal do not fully explain why females grunt.

Our data suggest that the mechanisms that underlie call production are in some respects similar to those that underlie listeners’ responses. Just as responses to calls depend on contextual factors like the caller’s identity and the nature of the listener’s recent interactions with the caller, the caller’s decision to call or remain silent depends upon the caller’s assessment of current circumstances like the presence of an infant, the caller’s and listener’s ranks, and the quality of the relationship between listener and caller. These assessments do not require that callers recognize mental states like anxiety in others; the decision to call or not could easily be shaped through learned contingencies. But such contingencies are also varied, because they rely on the caller’s assessment of what contextual factors are relevant to call production and what factors are not. Despite their limited vocal repertoire, baboons appear to be skilled at modifying call production in different social contexts.
